# Numerical Study of the Time–Periodic Electroosmotic Flow of Viscoelastic Fluid through a Short Constriction Microchannel

**DOI:** 10.3390/mi14112077

**Published:** 2023-11-08

**Authors:** Jianyu Ji, Shizhi Qian, Armani Marie Parker, Xiaoyu Zhang

**Affiliations:** Department of Mechanical and Aerospace Engineering, Old Dominion University, Norfolk, VA 23529, USA; jji016@odu.edu (J.J.); shizhi@gmail.com (S.Q.); aparker@nps.k12.va.us (A.M.P.)

**Keywords:** electroosmosis, AC electric field, characteristic frequency, elastic instability, non-Newtonian fluid, Oldroyd-B model

## Abstract

Electroosmotic flow (EOF) is of utmost significance due to its numerous practical uses in controlling flow at micro/nanoscales. In the present study, the time–periodic EOF of a viscoelastic fluid is statistically analyzed using a short 10:1 constriction microfluidic channel joining two reservoirs on either side. The flow is modeled using the Oldroyd-B (OB) model and the Poisson–Boltzmann model. The EOF of a highly concentrated polyacrylamide (PAA) aqueous solution is investigated under the combined effects of an alternating current (AC) electric field and a direct current (DC) electric field. Power-law degradation is visible in the energy spectra of the velocity fluctuations over a wide frequency range, pointing to the presence of elastic instabilities in the EOF. The energy-spectra curves of the velocity fluctuations under a DC electric field exhibit peaks primarily beneath 20 Hz, with the greatest peak being observed close to 6 Hz. When under both DC and AC electric fields, the energy spectra of the velocity fluctuations exhibit a peak at the same frequency as the AC electric field, and the highest peak is obtained when the frequency of the AC electric field is near 6 Hz. Additionally, the frequency of the AC electric field affects how quickly the viscoelastic EOF flows. Higher flow rates are obtained at relatively low frequencies compared to under the DC electric field, and the greatest flow rate is found close to 6 Hz. But as the frequency rises further, the flow rate falls. The flow rate falls to a level below the DC electric field when the frequency is sufficiently high.

## 1. Introduction

The invention of microfluidic devices and their applications in microelectromechanical systems has made it possible to organize and combine the processes of sample handling, detection, and analysis in integrated microfluidic platforms. In these applications, such as drug-delivery systems, biochemical analysis, rapid-mixing tools, and microelectromechanical systems [1,2,3], where various biofluids, such as blood, saliva, DNA suspensions, cerebrospinal fluid, lymphs, and proteins [4,5] are frequently used, electroosmosis flow (EOF) plays a critical role in controlling the movement of fluids in the microscale. These fluids exhibit a variety of sophisticated non-Newtonian behaviors, many of which have been linked to the fluids’ viscoelastic properties. Researchers have researched EOF in great detail since Reuss’s initial investigation [6]. However, the steady EOF of Newtonian fluids in various microcapillary geometric domains, such as the slit parallel plate [7,8], cylindrical capillary [9,10], T-shape [11], rectangular [12,13], annulus [14,15], semicircular [16], and sector [17] microchannel cross-sections, have been the main focus of EOF research. There has been relatively little research done on the EOF of non-Newtonian fluids, especially viscoelastic fluids [18,19,20,21,22,23]. And, just recently, experimental and numerical research revealed that the EOFs of non-Newtonian fluids are time-dependent and exhibit instabilities.

In actuality, a direct current (DC) electric field is employed in the EOF to drive the fluid in microchannels. The EOF of Newtonian fluids under a DC electric field is time-independent and in a steady state. The EOF of complex viscoelastic fluids, however, is only stable when the Weissenberg number, Wi, (which is a dimensionless number evaluating the relative importance of the elastic and viscous forces in the flow, and defined as the product of the relaxation time and the characteristic strain rate) is very low, because of the existence of extra elastic stress in viscoelastic fluids. The term “electro-elastic instability” (EEI) refers to the fluctuations in the EOF caused by the strong elastic effect when the Wi is relatively high (≈1, which also depends on factors such as geometry and fluid rheology). Bryce and Freeman [24] conducted the first experimental study on the EEI in the EOF of polyacrylamide (PAA) solutions using a constriction microchannel. Afonso et al. [25] first numerically reported the flow transition from a steady state to an unsteady state in the EOF of Maxwell fluid in a cross-slot geometry. Later, Pimenta and Alves [26,27] investigated the elastic instabilities in the EOF of PAA solution in the same cross-slot geometry using numerical and experimental methods, and they came to the conclusion that the instabilities were primarily induced by the shear-dominated flow within the electrical double layer (EDL) at the corners of the geometry as opposed to the extensionally dominated bulk flow. However, Song et al.’s [28] investigation on the influence of fluid rheology on the elastic instabilities discovered that the shear thinning effect, not high elasticity alone, accounts for the elastic instabilities. Through contraction microchannels, Sadek et al.’s [29] experimental examination of the viscoelastic EOF revealed instabilities of an elastic origin at a very-low Wi (i.e., Wi < 0.01). The instabilities in the EOF of viscoelastic fluid through a 10:1:10 contraction/expansion microchannel were found to be substantially dependent on the concentration of the polymers and the strength of the electric field in our earlier computational work [30]. Recent research by Datta et al. [31] provided a thorough overview and discussion of the characteristics and mechanisms of elastic instabilities in pressure-driven flow. However, the EOF’s plug-like velocity profile results in a significantly stronger shear impact within the EDL than the pressure-driven flow, which could result in variations in how the instabilities in the flow are triggered even under similar flow conditions. Khan et al. [32] recently reported the instability in electroosmotic flows of viscoelastic fluids through a model porous system, where the flow was observed to transit from steady to unsteady at a critical value of the Wi, and the critical Wi was related to the polymer viscosity ratio and expansion and contraction length of the micropore. There are extremely few studies that correspond to the elastic instabilities in the EOF.

Due to the bubble-formation issue in the electrolytes near the electrodes that the DC electric field experiences, periodic EOF is gaining more and more attention as a substitute for microfluidic control and transport in the microchannels. It has been discovered that some natural chemical processes, such as the electroosmosis of the human epidermal membrane [33], are associated with periodic EOF. Periodic electroosmosis, in contrast to steady electroosmosis, has a velocity profile that wavers with time and has been used to improve fluid mixing and flow-rate control in microchannels [34,35]. Dutta and Beskok [36] were among the early researchers investigating the time–periodic EOF between two parallel plates. Their analytical solution of velocity revealed near-wall inflection points with localized velocity extrema, which may have an impact on the stability properties of the time–periodic EOF. Ramos et al.’s theoretical investigation [37] has demonstrated that the EOF is frequency-dependent, with the velocity being a function of both space and frequency across microelectrodes. The experimental research of Minor et al. [38] on the electromobility of colloidal particles revealed that the bulk electroosmosis could be stopped by applying an AC electric field with a specific frequency. Microfluidic pumps based on AC–EOF have been the subject of research studies since the discovery of the AC–EOF conveyance mechanism. Studer et al. [39] fabricated microfluidic AC–EOF pump, and the velocity was found to be dependent on the frequency of the electric field. Within a wide range of frequencies from 0.1 kHz to 100 kHz, the maximum velocity of the pump was seen at 5 kHz. According to Olesen et al.’s experimental investigation [40,41], AC electrokinetic micropumps are more effective at pumping microflows when the driving voltage is low.

Newtonian fluids were the primary focus of all the pulsating EOF research work mentioned above. With only a small body of literature, the impact of periodic EOFs on non-Newtonian fluids is not well understood. The analytical solutions for the time–periodic EOF of general Maxwell fluids between micro parallel plates were initially examined by Liu et al. [42] utilizing the separation of variables approach, where the velocity profile and volume flow rate showed a strong dependence on the flow parameters. The analytical solution for the analogous flow in the rectangular microchannel was researched by Jian et al. [43]. Recent research by Moghadam and Akbarzadeh [44] into the time–periodic EOF of power-law fluid in a circular microchannel revealed that the flow was constrained to a thin region near the channel wall at extremely high dimensionless frequencies. Later, a study based on the Carreau–Yasuda model, which combines the pressure gradient and time–periodic electroosmosis, was also carried out by the same author [45]. The improved mixing effect of the AC–EOF in a T-junction micromixer was examined using a power-law model in Alipanahrostami and Ramiar’s numerical work [46]. Based on the PTT model, Sayantan and Sandip [47] employed the time–periodic EOF to regulate the mass flow rate of viscoelastic fluids. It was discovered that the mass flow rate amplitude and the phase lag were connected to the fluid’s viscoelastic properties. Dongsheng and Kun [48] investigated the time–periodic pulse electroosmotic flow of Jefferey fluids in a circular microchannel. The time for the flow to reach a steady state was affected by the pulse width and the retardation time, and the velocity amplitude was influenced by the density ratio and viscosity ratio. Vishal et al. [49] studied the combined electromechanically driven pulsating flow of nonlinear viscoelastic fluids in narrow confinements based on the PTT model. The extent of augmentation in the flow rate was found to be strongly dependent on the frequency and waveform of the driving force.

The mechanism of the time–periodic EOF of viscoelastic fluids is still unknown due to the relatively few investigations that have been undertaken. Therefore, the time–periodic EOF of viscoelastic fluids is investigated in the current study using 10:1:10 contraction microchannels. The viscoelastic properties of the PAA water solution are simulated using the OB model, and the electrokinetic phenomenon is described using the Poisson–Boltzmann (PB) model. The microchannel is subjected to both a continuous DC electric field and AC electric fields with different frequencies. The effects of the AC electric field frequency on the viscoelastic time–periodic EOF are investigated.

## 2. Mathematical Model

**Figure 1:**
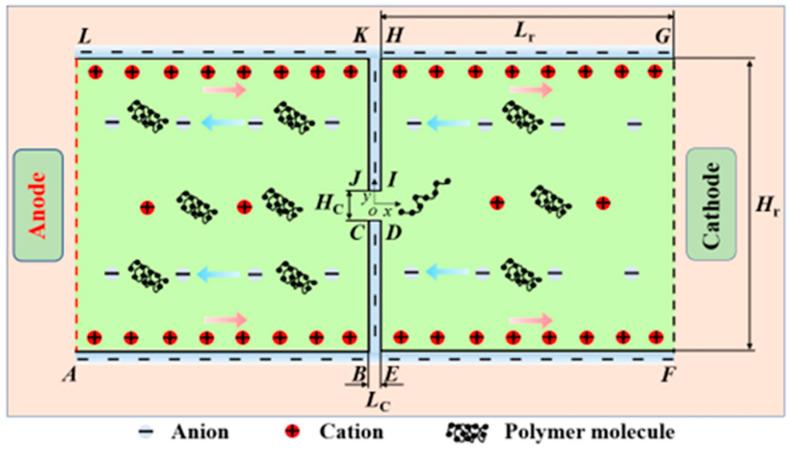
Schematic diagram of a constriction microchannel connecting two reservoirs at both ends.

We assume that the incompressible PAA polymer solution of concentration *c*_p_ is mixed with the electrolyte solution that has a bulk concentration of *c*_0_ and contains ions such as K^+^ and Cl^−^. The computational domain’s dimensions are depicted in Figure 1 as two identical reservoirs with height *H*_r_ and length *L*_r_ on either side of a short microchannel with height *H*_C_, length *L*_C_, and width W. Researchers have generally acknowledged that the zeta potential in the EOF of non-Newtonian fluids is constant [50]. As a result, the solid walls of the constriction microchannel and the reservoirs are assumed to carry a constant negative zeta potential, *ξ*_0_. Two electrodes are placed at both ends of the reservoirs, and an external potential bias *U* = *U*_0_ + *U*_A_sin(2*f*_E_π*t*) is applied between the inlet (Anode) and outlet (Cathode). The apparent electric field between the inlet and outlet is defined as *E*_app_ = *U*/(2*L*_r_ + *L*_C_). We assume that the channel width is significantly larger than the channel height and length, and the flow can be simplified to a 2D problem, as schematically shown in Figure 1. A Cartesian coordinate system is used, with the origin fixed at the center of the microchannel, with the *x*-axis running along the length direction and the *y*-axis running along the height direction.

The governing equations of the induced viscoelastic EOF are given as follows:

(1) ∇·u=0,

(2) $$\rho \left(\frac{\partial u}{\partial t}+u\cdot \nabla u\right)=-\nabla p+\eta_{s}\nabla^{2}u+\nabla \cdot \tau -\rho_{E}\nabla \phi_{Ext},$$

where ***u*** is the velocity field; *p* is the pressure; *t* is the time; *ρ* is the fluid density; *η*_s_ is the solvent dynamic viscosity; ***τ*** is the extra polymeric stress tensor; *ρ*_E_ represents the volume charge density in the electrolyte; *ϕ*_Ext_ represents the externally applied electric potential. For different types of viscoelastic fluids, the polymeric stress ***τ***, which accounts for the deformation memory of the fluid, can be described by various constitutive models. The most widely used viscoelastic models include the OB model [51] for dilute polymer solutions, the Glesekus model [52] for concentrated polymer solutions, the White–Metzner model [20] describing shear-thinning fluids, and the PTT model [53] capturing viscoelastic fluid rheology under low-shear rates. Among all of the available modules for viscoelastic fluids, the OB model has been proven to be a good fit for the rheology properties of aqueous PAA solutions [54]. Therefore, in the current study, the OB model is adopted, where ***τ*** is described as [51],

(3) $$\tau =\frac{\eta_{p}}{\lambda }(c-I),$$

where *η*_p_ is the polymer dynamic viscosity; λ is the relaxation time of the polymer describing the time for polymer chains to return to equilibrium after deformation; ***c*** is the polymeric conformation tensor; ***I*** is the identity matrix. For the OB model, the conformation tensor ***c*** is governed by [51],

(4) $$\frac{\partial c}{\partial t}+u\cdot \nabla c=c\cdot \nabla u+{\left(\nabla u\right)}^{T}\cdot c-\frac{1}{\lambda }(c-I).$$

Typically, the numerical simulation of viscoelastic flow is difficult to converge for the high-Weissenberg-number problem (HWNP) [55,56]. To solve this problem, the log-conformation tensor approach [56] is adopted, ensuring the positive definiteness of the conformation tensor, which is essential for the numerical stability for the HWNP. A new tensor (**Θ**) is defined as the natural logarithm of the conformation tensor,

(5) $$\Theta =\ln\left(c\right)=R\ln\left(\Lambda \right)R^{T},$$

where **Λ** is a diagonal matrix, whose diagonal elements are the eigenvalues of ***c***, and **R** is an orthogonal matrix with its columns being the eigenvalues of ***c***. Equation (4) for the conformation tensor written in terms of **Θ**, then becomes [55],

(6) $$\frac{\partial \Theta }{\partial t}+u\cdot \nabla \Theta =\Omega \Theta -\Theta \Omega +2B+\frac{1}{\lambda }(e^{\Theta }-I).$$

In the above, **Ω** and **B** are, respectively, the antisymmetric matrix and the symmetric traceless matrix of the decomposition of the velocity gradient tensor ∇***u*** [55].

Then, the conformation tensor ***c*** is recovered from **Θ**,

(7) c=exp(Θ).

The total electric potential, *Ψ*, is decomposed into two variables, *Ψ* = *ϕ*_Ext_ + *ψ* [27], where *ϕ*_Ext_ represents the potential originating from the externally applied electric potential and *ψ* is the potential arising from the charge distribution inside the EDL. The EDL thickness is dependent on the bulk ion concentration. A larger bulk ion concentration leads to a smaller EDL and more refined mesh near the walls to resolve the local wall-normal gradients accurately. To save computational cost, a relatively small bulk ion concentration is adopted in the current study. However, the EDL thickness is still on the order of nanometers, which is much smaller than the microchannel height. Therefore, the Poisson–Boltzmann equation [57] is used to describe the potential, *ψ*:

(8) $$\nabla \cdot \left(\varepsilon \nabla \psi \right)={-\rho }_{E}=Fc_{0}(\exp\left(\frac{e\psi }{kT}\right)-\exp\left(-\frac{e\psi }{kT}\right)).$$

In the above, *F* is the Faraday’s constant (i.e., 96,485.33289 C∙mol^−1^); *e* is the elementary charge (i.e., 1.6021766341 × 10^−19^ C); *k* is Boltzmann’s constant (i.e., 1.380649 × 10^−23^ J∙K^−1^); *T* is the absolute temperature of the fluid (i.e., 295 K); *ε* represents the permittivity of the solution (i.e., 6.906266 × 10^−10^ F∙m^−1^); *c*_0_ is the bulk ion concentration (i.e., 0.0001 mM). The EDL thickness can then be calculated by $\lambda_{D}=\sqrt{\frac{\varepsilon kT}{eF(z_{1}^{2}c_{0}+z_{2}^{2}c_{0})}}$ (i.e., 954 nm), in which z_1_ = 1, and z_2_ = −1. The dimensionless Debye number is defined as $\tilde{\kappa }=\frac{H_{c}}{\lambda_{D}}$. In this study, $\tilde{\kappa }\approx 42$. It should be noted that the *c*_0_ was selected to have *λ*_D_ affordable for numerical simulations. The study of Afonso et al. [25] showed that, when $\tilde{\kappa }>20$, the results are nearly independent of $\tilde{\kappa }$. Moreover, in the study of Pimenta et al. [27], a similar bulk concentration *c*_0_ = 0.000091 mM was used, and the numerical results showed agreement with experimental results. Therefore, we consider that $\tilde{\kappa }\approx 42$ is sufficient to avoid EDL overlapping and guarantee the accuracy. The potential *ϕ*_Ext_ is governed by the following Laplace equation [58],

(9) $$\nabla^{2}\phi_{Ext}=0.$$

The boundary conditions are given as follows:

(1) At the Anode (edge AL in Figure 1): **n**∙∇***u*** = 0; *p* = 0; ***τ*** = **0**; *ϕ*_Ext_ = *U*_0_ + *U*_A_sin(2*f*_E_π*t*); **n**∙∇*ψ* = 0; **Θ** = **0**; where **n** denotes the normal unit vector on the surface;
(2) At the Cathode (edge FG in Figure 1): **n**∙∇***u*** = 0; *p* = 0; **n**∙∇***τ*** = 0; *ϕ*_Ext_ = 0; **n**∙∇*ψ* = 0; **n**∙∇**Θ** = 0;
(3) On the reservoir walls (edges ABC, DEF, GHI, and JKL in Figure 1) and the microchannel walls (edges CD and IJ in Figure 1): ***u*** = **0**; **n**∙∇*ϕ*_Ext_ = 0 *ψ* = *ξ*_0_; and **n**∙∇**Θ** = 0.

The following initial conditions are specified within the domain: ***u*** = **0**; *p* = 0; ***τ*** = **0**; *ϕ*_Ext_ = 0; *ψ* = 0; **Θ** = **0**.

Note that the electric potentials and the flow are only one-way coupling. The electric potentials *ϕ*_Ext_ and *ψ* are independent on the flow. However, the electric potentials affect the flow through the electrostatic force, which is the last term in Equation (2). For Newtonian fluid, the third term, ∇∙***τ***, in the right-hand side of Equation (2) is dropped, and the model includes Equations (1), (2), (8) and (9).

## 3. Numerical Method and Code Validation

The governing equations are numerically solved using an open-source framework, OpenFOAM [59], integrated with the viscoelastic solver RheoTool [26] (version 5.0, https://github.com/fppimenta/rheoTool). The equations are discretized in OpenFOAM using the finite-volume method. The details of the viscoelastic solver can be found in the work of Pimenta and Alves [26,58]. A summary of the main features of the solver is as follows. The convective terms in Equations (2) and (6) are discretized using the CUBISTA (Convergent and Universally Bounded interpolation Scheme for Treatment of Advection) scheme [60]. Central differences are used for the discretization of Laplacian and gradient terms. The Euler time-integration scheme is used to discretize the time derivatives [61], which is of the second order of accuracy. The exponential source term in Equation (8) is linearized using Taylor expansion up to the second term [62]. All the terms in the momentum equation (i.e., Equation (2)), except the pressure gradient and the electric contribution, are discretized implicitly. A small timestep, ∆*t* = *λ*/10^5^, is used to ensure accuracy. The well-known SIMPLEC (Semi-Implicit Method for Pressure-Linked Equations–Consistent) algorithm [63] is used to resolve the velocity–pressure coupling. An inner-iteration loop is used to reduce the explicitness of the method and increase its accuracy and stability. The pressure field is computed by the PCG (Preconditioned Conjugate Gradient) solver, of which the tolerance and maximum iteration are set to be 1 × 10^−8^ and 800, respectively. The velocity field is computed by the PBiCG (Preconditioned Biconjugate Gradient) solver, of which the tolerance and the maximum iteration are set to be 1 × 10^−10^ and 1000, respectively.

**Figure 2:**
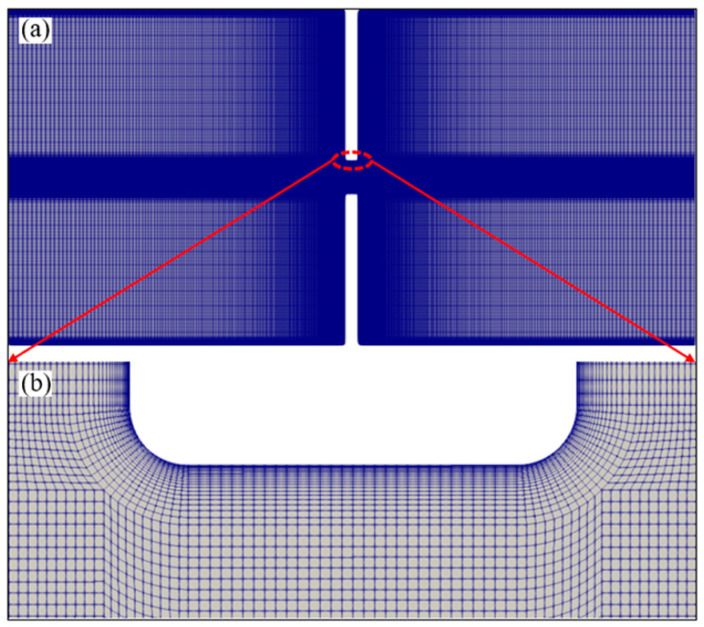
Computational mesh used in the numerical simulations for microchannels with a length of 15 µm. (**a**) Mesh of the whole geometry and (**b**) detailed view of the mesh at the constriction microchannel.

The structural mesh used to discretize the 2D computational domain is shown in Figure 2. The geometry at 90° corners is slightly rounded to avoid the electric-field singularity [64]. In order to capture the steep gradients in the vicinity of the charged walls, at least 10 cells need to be generated within the thin EDL for the modeling of the EOF. Therefore, as seen in Figure 2b, finer mesh is spread close to the charged reservoir and channel walls. To reduce the number of mesh, we use a relatively low bulk concentration *c*_0_ *=* 0.0001 mM, and the EDL thickness is about 954 nm in this study. The edge size of the layer of cells adjacent to the walls is 25 nm, and there are 15 cells in the EDL. Contraction microchannels with three different lengths (i.e., 10 µm, 15 µm, and 20 µm) are investigated in this study. As shown in Figure 2, the mesh for the 10 µm microchannel has a total of 526,560 cells, and there are 530,780 and 535,000 cells for the 15 µm microchannel and 20 µm microchannel, respectively. A mesh-independence study, described in the Appendix A, is performed for the 15 µm microchannel.

In this work, *η*_p_ = 0.0111 kg/(m∙s) and *λ* = 0.0476 s were adopted for the 500 ppm PAA–water solution, which were experimentally measured [65], and the slow-retraction method was used to measure the relaxation time. The zeta potential *ξ*_0_ is −110 mV. The solvent dynamic viscosity *η*_s_ is 0.001 kg/(m∙s). The external potential bias is *U* = *U*_0_ + *U*_A_sin(2*f*_E_π*t*), and the apparent electric field is defined as *E*_app_ = *U*/(2*L*_r_ + *L*_C_) = *E*_0_ + *E*_A_sin(2*f*_E_π*t*). In the current study, *L*_r_ = *H*_r_ = 400 µm; *H*_C_ *=* 40 µm; *L*_C_ = 10 µm, 15 µm, and 20 µm; *U*_0_ = 40 V; *U*_A_ = 16 V; the range of *f*_E_ is investigated from 0 Hz to 20 Hz. In order to quantify the viscoelastic effect of the EOF, the Weissenberg number (*Wi*) is calculated in this study, which is defined as *Wi* = *λu/H*_C_. Here, is the height of the microchannel, and u is the cross-sectional averaged velocity at the center of the microchannel (*x* = 0). In this study, the *Wi* is between 1 and 3 depending on the velocity.

A thorough code validation of the solver was carried out in our earlier study [30], including the comparison of the OB model and Newtonian fluids in a 10:1:10 contraction/expansion straight microchannel when the elastic effect is negligible, and the comparison between the Debey–Hückel approximation-based analytical solution of Afonso et al. and the numerical results of the viscoelastic EOF between two parallel plates. When the elastic effect is negligible, the relative difference between Newtonian fluids and the OB model is less than 0.5%, and the numerical result of the viscoelastic EOF is in excellent agreement with the analytical result. Furthermore, the solver is validated for solving the electroosmotic flow under pulsating electric fields, as described in Appendix B.

## 4. Results and Discussion

In this section, the flow features of the viscoelastic EOF through short constriction microchannels are obtained when imposed to constant and pulsating electric fields. Three different lengths (i.e., 10 µm, 15 µm, and 20 µm) of the microchannel are studied. Newtonian fluid is investigated for the 10 µm microchannel to provide reference flow characteristics. The frequency of the pulsating electric field is investigated from 1 Hz to 20 Hz. The instabilities of the viscoelastic EOF are described. The energy spectra of the viscoelastic EOF under a constant electric field shows a main frequency. Finally, the influence of the frequency of the pulsating electric field on the viscoelastic EOF is analyzed.

### 4.1. Characteristic Frequency of the EOF under a Constant Electric Field

**Figure 3:**
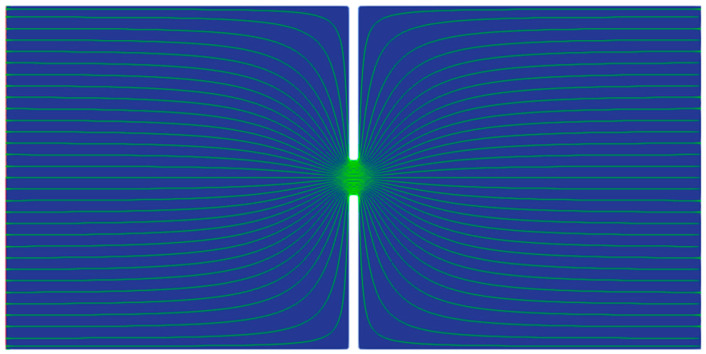
Time-independent streamlines of Newtonian fluid under the DC electric field for the 10 µm microchannel.

**Figure 4:**
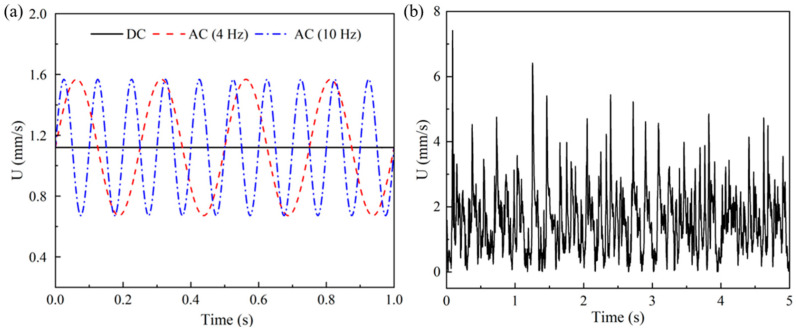
Velocity at the center of the 10 µm microchannel (i.e., (0,0)): (**a**) Newtonian fluid under the DC electric field and the AC electric field (f_E_ = 4 Hz and 10 Hz); (**b**) 500 ppm PAA solution under the DC electric field.

The results of Newtonian fluid with the same total viscosity as the 500 ppm PAA solution are first presented as a reference for the viscoelastic fluid. Under the DC electric field, the EOF reaches a steady state for Newtonian fluids. As shown in Figure 3, the streamlines of Newtonian fluid show excellent symmetry about the *x*-axis and *y*-axis. Figure 4a shows the velocities at the center of the microchannel (i.e., (0,0)) for the Newtonian fluid under the DC electric field and AC electric fields (*f*_E_ = 4 Hz and 10 Hz). Under the DC electric field, the velocity is time-independent, and no fluctuation is observed. Under the AC electric field, the velocity fluctuates at the same frequency as the electrical driving force. No minor fluctuation is observed in the velocity other than the fluctuation in the main frequency. Under different frequencies, the amplitude of the velocity fluctuation and the cross-sectional time-averaged velocity show no significant difference. However, for the PAA solution, the velocity at the center of the microchannel fluctuates dramatically even under the DC electric field, as shown in Figure 4b.

**Figure 5:**
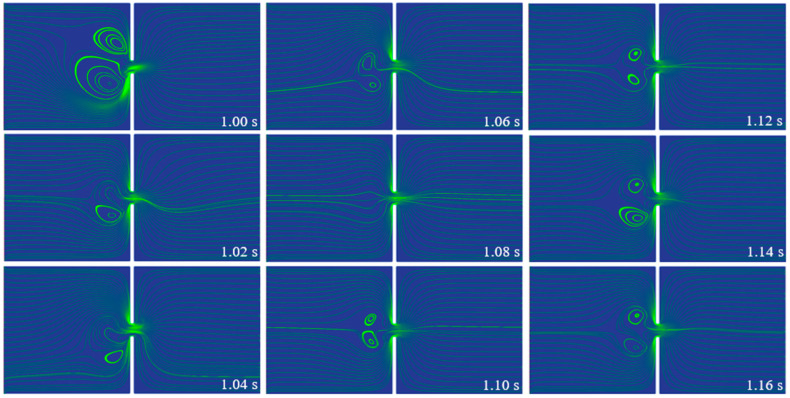
Streamlines of the 500 ppm PAA solution in the 10 µm microchannel under the DC electric field.

**Figure 6:**
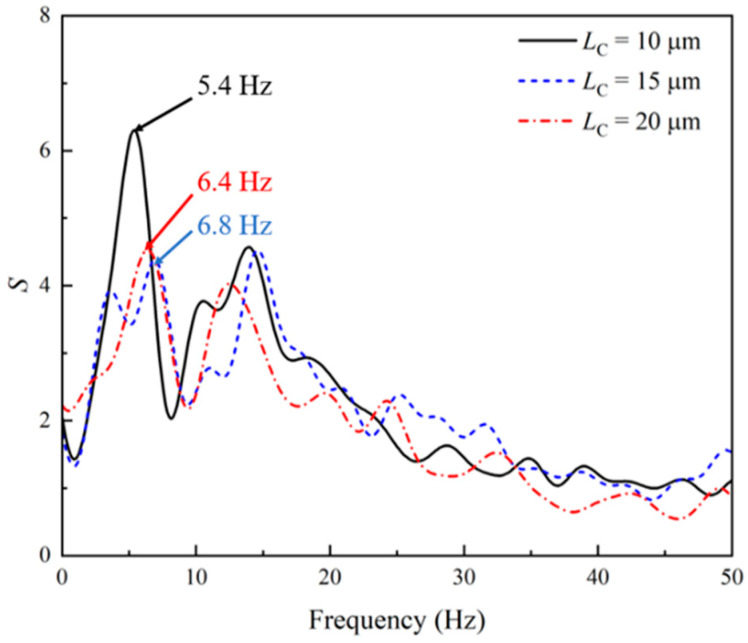
Energy spectra of the velocity fluctuation in the viscoelastic EOF at the center of the microchannel (i.e., (0,0)) when under the DC electric field.

Figure 5 displays the streamlines of the PAA solution at different times when subjected to the DC electric field. Different from the steady flow state of the Newtonian EOF, the viscoelastic EOF is time-dependent. The strong fluctuation of the velocity field is observed in the left inlet reservoir near the entrance of the constriction microchannel. A pair of unstable vortices are observed to fluctuate in both size and location. As shown in Figure 5, from 1 s to 1.08 s, the size of the vortices gradually decreases until the vortices disappear at 1.08 s, after which the vortices form again and grow in size. At 1 s, the pair of vortices is oriented to the direction above the centerline of the microchannel. However, at 1.04 s, an opposite orientation of the pair of vortices is observed. Due to the existence of the stagnation area and the fluctuation of vortices orientation, the flow along the centerline in the right outlet reservoir is affected and fluctuates. Similar vortices were reported and analyzed in our earlier numerical study of a long constriction microchannel [30], where the instabilities in the viscoelastic EOF were found to be caused by the extra elastic stress and the curvature of the streamline. Unlike Newtonian fluids, the existence of elasticity is a unique feature of viscoelastic fluids, which results in the existence of a characteristic frequency (*f*_C_) in the viscoelastic flow. In order to have a deeper understanding of the instabilities observed in the viscoelastic EOF under the DC electric field, the power spectra of the velocity fluctuation are obtained by conducting Fast Fourier Transformation. Figure 6 shows the kinetic energy spectra of the velocity fluctuations at the center of the microchannel (i.e., (0,0)) in the frequency domain. For the microchannel with three different lengths, the energy-spectra curves exhibit similar characteristics. The majority of the energy of the velocity fluctuation is distributed in the range 0–20 Hz. Under the DC electric field, a clear dominant frequency between 5 Hz and 7 Hz is observed in the energy spectra of the velocity fluctuations, which represents the characteristic frequency of the viscoelastic flow. Such characteristic frequency of the viscoelastic flow is also referred to as the resonance frequency in the dynamics of polymeric viscoelastic solutions. The flow of viscoelastic solutions is strongly affected by many factors, including the chemical properties of the polymer, the molecular weight, the concentration, solvent, temperature, flow geometry, etc. [66]. Although limited studies have been performed for the electroosmotic flow, the resonance frequency of the viscoelastic flow has been studied in pressure-driven flow both analytically and experimentally [67,68,69], where the resonance frequency was defined as the frequency under which the flow showed the highest dynamic permeability based on observation. However, due to the complexity of viscoelastic fluids, and the mostly used confined flow condition, no general method for the direct prediction of the resonance frequency of the viscoelastic flow has been proposed. It has been commonly accepted that such resonance frequency is related to the elasticity and relaxation time of viscoelastic fluids, as well as the flow geometry. For example, the study of Yu et al. [70] showed that the first resonance frequency of Maxwell fluid in a tube showed dependency on a dimensionless radius, which was calculated based on the radius of the tube and the relaxation time. Moreover, in the study of Collepardo and Poire [68], the resonance frequency of Maxwell fluid was found to shift with the change in obstructions. In the experimental study of Pamela et al. [69], the resonance frequency of viscoelastic fluids was dependent on the magnitude of the pressure drop. Therefore, the characteristic frequency for the viscoelastic EOF is expected to shift with the changes in the fluid properties and the flow geometry. Despite the different approaches, one common application in the studies of the pulsating pressure-driven flow of viscoelastic fluids is flow-enhancement or flow-tuning. However, relative resonance studies of the electroosmotic flows of viscoelastic fluids are very limited.

### 4.2. Frequency Study of the Viscoelastic EOF under a Pulsating Electric Field

In order to investigate the effects of the characteristic frequency on the viscoelastic EOF, a pulsating electric field is applied to the microchannel. The energy spectra of the velocity fluctuation under the DC electric field show that the majority of the energy of the fluctuation is distributed under 20 Hz in the frequency domain, and the characteristic frequency of the viscoelastic EOF is between 5 Hz and 7 Hz. Therefore, the frequency of the externally applied electric field is studied from 1 Hz to 20 Hz. The velocity at the center of the microchannel (i.e., (0,0)) is used for the frequency analysis. As shown in Figure 4a, in the Newtonian pulsating EOF, the amplitude of the velocity keeps constant for different pulsating frequencies. However, in the viscoelastic pulsating EOF, the amplitude of the velocity and the cross-sectional time-averaged velocity show a significant difference when under the AC electric field with different frequencies.

**Figure 7:**
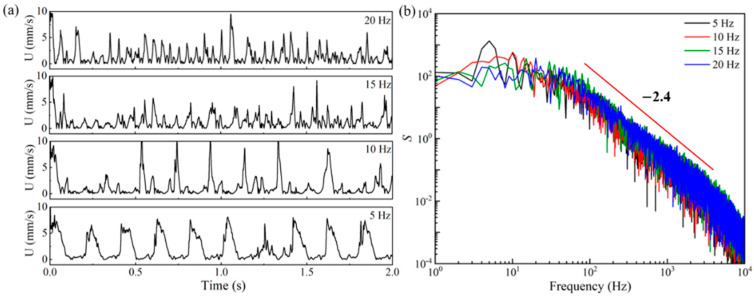
Velocity at the center of the 10 µm microchannel under the pulsating electric field with different frequencies (i.e., *f*_E_ = 5, 10, 15, and 20 Hz): (**a**) Velocity–time profile and (**b**) energy spectra of the velocity fluctuations.

Figure 7 displays the velocity and the energy spectra of the velocity fluctuation of the viscoelastic EOF at the center of the microchannel under the pulsating electric field with different frequencies (i.e., 5 Hz, 10 Hz, 15 Hz, and 20 Hz), where significant differences can be seen. As shown in Figure 7a, a distinct periodic velocity profile with relatively large and uniform velocity amplitudes is formed when the frequency of the pulsating electric field is 5 Hz, which is close to the characteristic frequency of the viscoelastic fluid. However, the velocity fluctuation is more erratic when the frequency of the electric field is higher than the characteristic frequency of the viscoelastic fluid. The magnitude of each peak in the velocity–time profile, for instance, varies significantly at 10 Hz. Within the first 2 s, seven relatively large peaks are observed, while other peaks are extremely small. For the velocities under the 15 Hz and 20 Hz electric field, the amplitudes of the velocity fluctuation are more uniform than under the 10 Hz electric field; however, the amplitudes are still generally much smaller than under the 5 Hz electric field. Figure 7b displays the energy spectra of the velocity fluctuations at the center of the microchannel to provide a more detailed understanding of the frequency distribution of the velocity fluctuation. For the viscoelastic EOF under pulsating electric fields with different frequencies, the power spectra curves share the same general appearance: there is a plateau at low frequencies followed by a power-law decay region at higher frequencies, which is a unique feature of elastic turbulence [71]. The exponent is around −2.4 for all the energy-spectra curves. In the power spectra of the velocity fluctuation under the 5 Hz pulsation electric field, a clear peak is observed at 5 Hz. However, for the energy-spectra curves under pulsating electric fields with higher frequencies, although the highest energy appears at the frequencies of the electric driving force, no significant peaks are observed in the rest-energy-spectra curves in Figure 7b compared with under the 5 Hz electric field.

**Figure 8:**
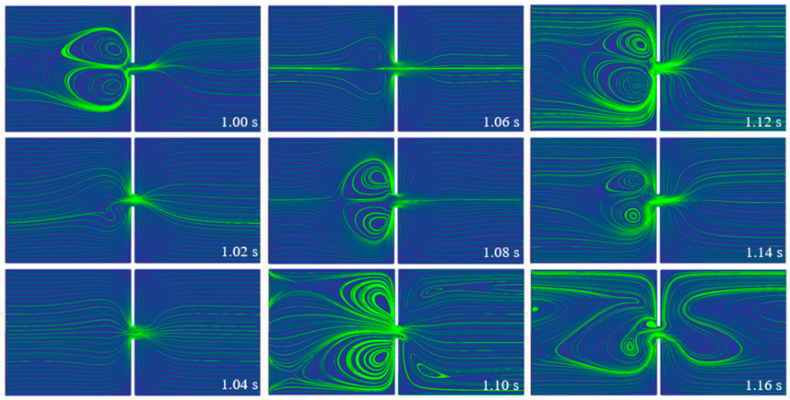
Streamlines of the 500 ppm PAA solution in the 10 µm microchannel under the 5 Hz pulsating electric field.

**Figure 9:**
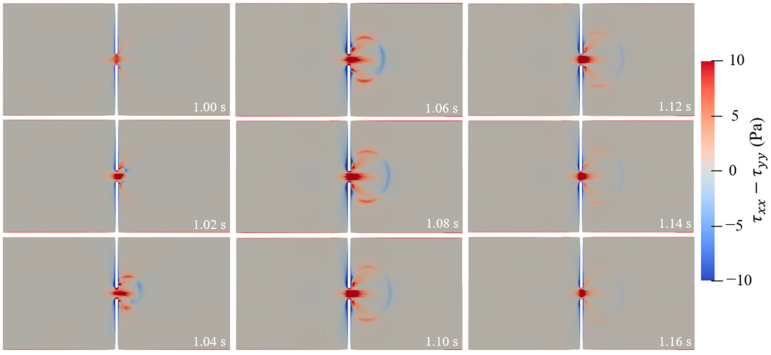
First normal stress difference of the 500 ppm PAA solution in the 10 µm microchannel under the 5 Hz pulsating electric field.

Figure 8 depicts the streamlines of the viscoelastic fluid in the 10 µm microchannel under the 5 Hz pulsating electric field. Compared with the streamlines under the DC electric field, as shown in Figure 5, more chaotic streamlines are observed under the pulsating electric field. At *t* = 1 s, a pair of large vortices are observed in the left inlet reservoir before the entrance of the constriction microchannel, resulting in a stagnation area along the centerline of the microchannel. Then, the pair of vortices vanish because of the shifting electric field; at *t* = 1.08 s, the vortices form again. However, at *t* = 1.10 s, the pair of vortices move away from the centerline of the microchannel to the corner of the left inlet reservoir, forming a stagnation in the flow near the upstream lips of the constriction microchannel, and the fluid at the centerline of the inlet reservoir flows through the constriction microchannel. Moreover, in the right outlet reservoir, two small vortices form near the downstream lips of the constriction microchannel. The upstream lip vortices are unstable and move to the centerline of the microchannel with the change in the pulsating electric field, as shown in the streamlines at *t* = 1.12 s and *t* = 1.14 s, resulting in the fluid near the charged walls of the inlet reservoir flowing through the constriction microchannel. At *t* = 1.16 s, the streamlines are even more chaotic, in which two small unsymmetric vortices form before the entrance of the constriction microchannel and lead to the fluid flowing through only the lower half of the microchannel. And in the outlet reservoir, the fluid flows near the charged walls, forming a large circulation near the centerline of the reservoir. The upstream lip vortices and the downstream vortices observed under the pulsating electric field are unique and not observed under the DC electric field. Figure 9 shows the first normal stress difference N1(*τ_xx_* − *τ_yy_*) of the 500 ppm PAA solution in the 10 µm microchannel under the 5 Hz pulsating electric field at different times. A significant positive increase of N1 is induced, which indicates that the polymer molecules are strongly stretched in the *x*-axis direction, whereas in the *y*-axis direction, the polymer molecules are relatively compressed. The increase of N1 is due to the high velocity gradient at the entrance of the microchannel. Due to the relaxation time of the polymer molecules, the stretched polymer molecules take time to relax. Therefore, the positive N1 is observed downstream of the constriction microchannel.

**Figure 10:**
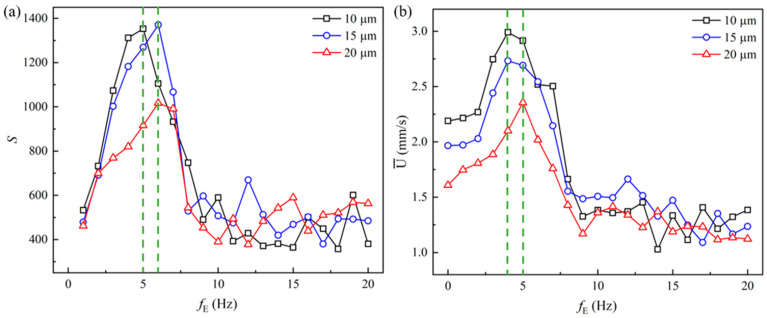
Statistical results of the viscoelastic EOF: (**a**) Magnitude of the energy spectra at the frequency of the pulsating electric field; (**b**) Cross-sectional average velocity. The green lines show the peak for each curve.

Figure 10 shows the statistical results of the viscoelastic EOF under the pulsating electric field with frequencies from 1 Hz to 20 Hz. Figure 10a plots the magnitude of the energy spectra at the dominant frequency, which is the frequency of the pulsating electric field. For the 10 µm microchannel, the highest energy magnitude appears at *f*_E_ = 5 Hz, while for the 15 µm and 20 µm microchannels, the highest energy magnitude appears at *f*_E_ = 6 Hz. Such results show consistency with the energy-spectra curves in Figure 6, which shows the characteristic frequency of the viscoelastic fluid under the DC electric field. When the pulsating electric field is at the same frequency as the characteristic frequency of the viscoelastic fluid, the magnitude of the energy spectra at the dominant frequency is much higher than when the pulsating electric field is at frequencies far from the characteristic frequency of the viscoelastic fluid, which indicates that the energy of the velocity fluctuation is highly distributed near where the characteristic frequency and resonance takes place. Figure 10b shows the cross-sectional average velocities of the three microchannels. Since the flow velocity is time-dependent, the cross-average velocity over a period of ∆*t* = *t*_2_ − *t*_1_ is calculated. The time-averaged velocity is calculated as:

(10) $$\bar{U}=\frac{\int_{t_{1}}^{t_{2}}\int_{-H_{c}/2}^{H_{c}/2}U(0,y)dydt}{∆t\cdot H_{c}},$$

where ∆*t* = 2 s is adopted in the current study. Intriguing characteristics of the statistical outcome of the average velocities can be seen in the following aspects: (1) The length of the microchannel shows significant influence on the pulsating viscoelastic EOF. The average velocity is generally higher in shorter microchannels. In the constriction microchannel, the polymer molecules are stretched in the *x*-axis direction and compressed in the *y*-axis direction. A shorter microchannel allows the molecules to pass through the channel faster, and thus relax faster. Therefore, a smaller resistance is experienced when the molecules flow through the microchannel, leading to a higher flow rate; (2) The average velocity in the microchannel is highly dependent on the frequency of the pulsating electric field, and the average velocity increases dramatically under the pulsating electric field with increasing *f*_E_ until *f*_E_ reaches a value close to the characteristic frequency of the viscoelastic fluid. However, with the further increasing of $f_{E}$, the average velocity decreases dramatically until *f*_E_ = 9 Hz; when *f*_E_ is relatively high (>9 Hz), the average velocity decreases to a level smaller than the average velocity under the DC electric field; (3) The highest average velocity is obtained when *f*_E_ = 4 Hz for the 10 µm and 15 µm microchannels, and the highest average velocity in the 20 µm microchannel is observed at *f*_E_ = 5 Hz, which are slightly smaller than the frequencies observed for the highest energy amplitude in Figure 10a. Such frequency-dependent EOFs have been reported in analytical studies of viscoelastic fluids [47,48]. More analytical and experimental studies of pulsating viscoelastic fluid flow have been performed based on pressure-driven flow [68,69,72,73]. Such a frequency-dependence of the flow velocity has been described as the resonance of the pulsating viscoelastic fluid flow, which is related to the elasticity and the inherent relaxation time of the viscoelastic fluid. More specifically, the coupling between the nonlinear constitutive structure of the viscoelastic fluid and the pressure leads to the flow-rate-enhancement effect in the pulsating pressure-driven flow [70]. It has also been proposed in the study of Siginer [74] that, in pressure-driven flow, the coupling of nonlinear elastic and shear-thinning properties causes sharp increases in the flow rate at lower frequencies, while the coupling of linear elastic and viscous properties induces flow-rate increases at relatively large frequencies. It should be noted that the shear-thinning effect is not included in the current study. However, due to the coupling between the conformation tensor and the polymeric stress in the constitutive equation, strong nonlinearity is introduced to the flow. Despite the difference of the driving force, a very similar flow-rate-enhancement effect is observed in current study as in the pressure-driven flow. Under the DC electric field, the viscoelastic EOF fluctuates at a dominant natural frequency between 5 Hz and 6 Hz. Under the AC electric field, when the frequency of the driving force is close to the natural frequency of the fluid, i.e., around 5 Hz, a resonance-like behavior is induced, which results in the dramatic increase in the energy spectra and the flow rate (represented by the average velocity). However, when the frequency of the driving force is much larger than the natural frequency of the fluid, instead of inducing a resonance-like behavior, the pulsating driving force implies a suppressive effect on the viscoelastic EOF. Thus, by applying the pulsating electric field, the flow rate can be controlled solely by the selection of the frequency without changing the amplitude of the electric field. However, more theoretical investigations remain to be performed to have a better understanding of the flow-rate-enhancement regime in the pulsating EOF of viscoelastic fluids with different properties. Even though pressure-driven pulsating-viscoelastic-fluid flow has demonstrated similar properties, the advantage of the pulsating EOF as an approach to control viscoelastic fluids is that the pulsating electric field is easier to apply and can prevent bubble-formation and the heating issues of the DC electric field [75].

## 5. Conclusions

The electroosmotic flow (EOF) of viscoelastic fluid through short 10:1:10 constriction microchannels was numerically investigated as a function of the frequency of the pulsating electric field. Three lengths of the constriction microchannel were studied. The frequency of the pulsating electric field was varied from 1 Hz to 20 Hz. The EOF of Newtonian fluid under the pulsating electric field was studied for reference. Compared with the Newtonian EOF and viscoelastic EOF under the DC electric field, the pulsating viscoelastic EOF shows the following distinct results:

(1) Under the DC electric field, the Newtonian EOF is time-independent. Under the pulsating electric field with the same amplitude, the amplitude of the velocity and the average velocity in the Newtonian EOF is independent of the frequency of the pulsating electric field. However, the viscoelastic EOF shows significant fluctuations under the DC electric field and strong dependence on the frequency of the pulsating electric field;
(2) For the viscoelastic EOF under the DC electric field, the dynamic energy spectra of the velocity fluctuation at the center of the microchannel viscoelastic EOF shows a dominant frequency, which indicates the existence of the characteristic frequency of the viscoelastic fluid;
(3) Under pulsating electric fields with various frequencies, strong instabilities are triggered in the viscoelastic EOF, with random upstream and downstream vortices observed. The energy-spectra curves of the velocity fluctuations share similar general features with a peak at the dominant frequency and a power-law decay over a wide range of frequencies, which is a typical characteristic of elastic turbulence;
(4) The highest magnitude of the energy spectra is observed at the frequency of the pulsating electric field. However, the highest magnitude varies with the exciting frequency, and resonance occurs in the EOF when the frequency of the pulsating electric field is near the characteristic frequency of the viscoelastic fluid observed under the DC electric field;
(5) The average velocity in the microchannel is highly dependent on the frequency of the pulsating electric field. When the frequency is relatively low, the average velocity increases with the increasing frequency, and the highest average velocity is observed near the characteristic frequency of the viscoelastic fluid. However, at relatively high frequencies, the average velocity decreases to a level even smaller than under the DC electric field.

## Figures and Tables

**Figure A1:**
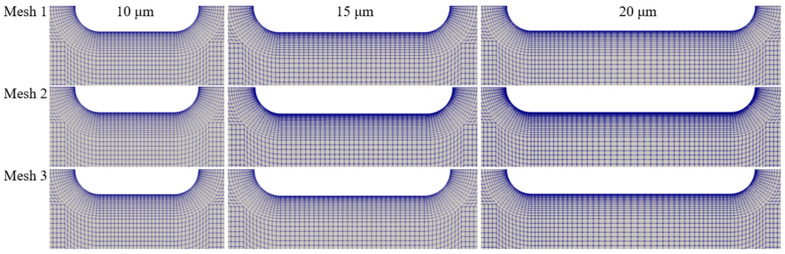
Detailed views near the charged wall of the three different meshes for the three microchannels used for the mesh-independence study.

**Figure A2:**
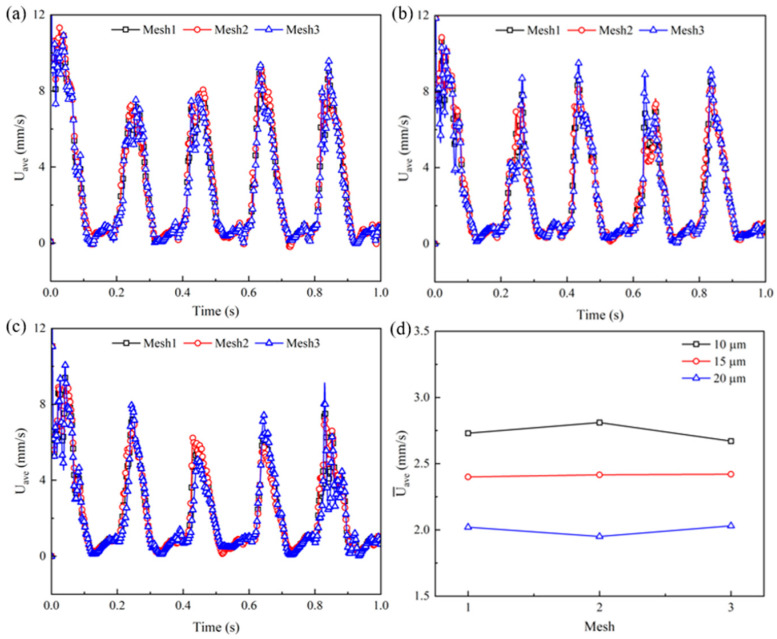
Cross-sectional average velocities at x = 0 in the microchannel: (**a**) Velocities in the 10 µm microchannel, (**b**) velocities in the 15 µm microchannel, (**c**) velocities in the 10 µm microchannel, (**d**) and time-averaged cross-sectional average velocities.

**Figure A3:**
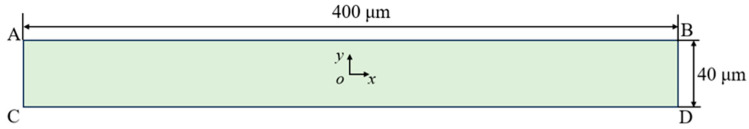
Straight microchannel for code validation.

**Figure A4:**
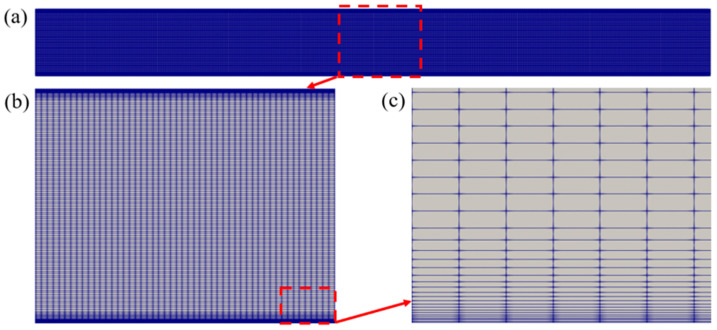
Computational mesh used in code validation. Mesh of the whole straight microchannel (**a**) and detailed view of the mesh along the cross section of the microchannel (**b**), and near the solid wall (**c**).

**Figure A5:**
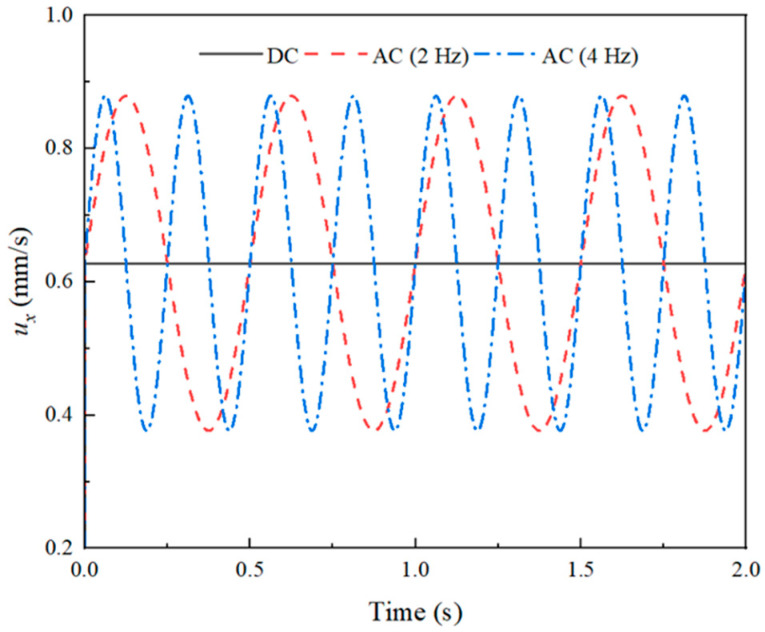
Velocity in the *x*-axis direction (*u_x_*) at the center of the microchannel (0,0) when *f*_E_ = 0, 2, and 4 Hz.

**Figure A6:**
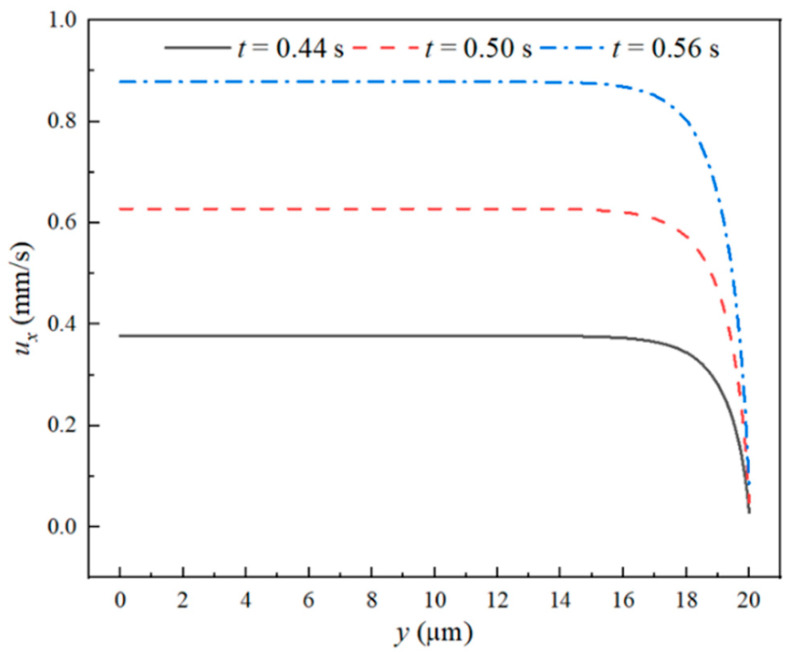
Velocity profile in the upper-half cross-section of the microchannel when *f*_E_ = 4 Hz, at *t* = 0.44 s, *t* = 0.50 s, and *t* = 0.56 s.

**Figure A7:**
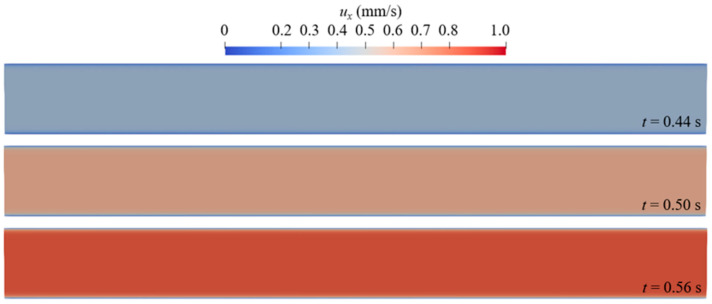
The *x*-component velocity field in the microchannel when *f*_E_ = 4 Hz at *t* = 0.44 s, *t* = 0.50 s, and *t* = 0.56 s.

**Figure A8:**
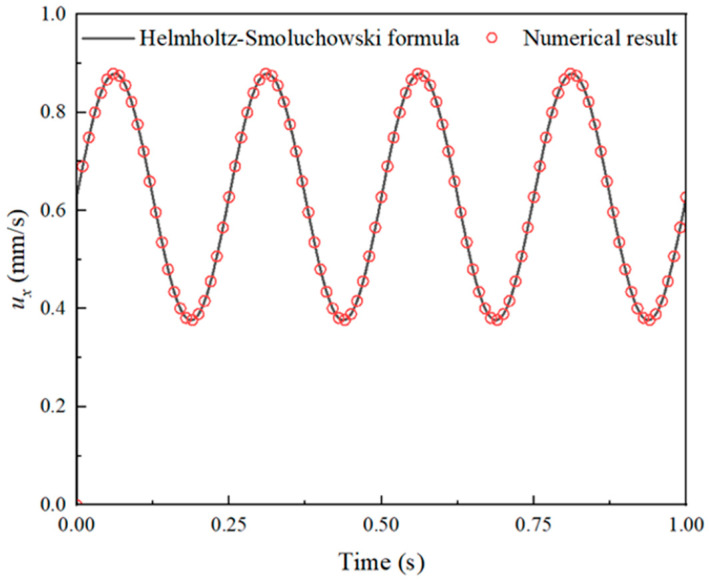
The *x*-component velocity at the center of the microchannel (0,0). Helmholtz–Smoluchowski velocity formula (solid line) and numerical result (symbol).

## Data Availability

Data is unavailable due to privacy.

## Appendix A. Mesh-Independence Study

Three different meshes are used to perform a mesh-independence study for each microchannel for the 500 ppm PAA solution, as shown in Figure A1. For mesh 1, the meshes near the charged walls are 25 nm, and there are 15 cells within the EDL thickness. In mesh 2, there are 20 cells within the EDL thickness, and the size of the cells near the charged walls is 20 nm. The cells outside the EDL thickness are the same for mesh 1 and mesh 2. In mesh 3, the cells within the EDL thickness are the same as in mesh 1. However, the cells outside the EDL thickness are slightly finer than mesh 1. The number of cells in each mesh for the three microchannels is listed in Table A1.

**Table A1:** Number of cells in each mesh used for the mesh-independence study.

Number of Cells	10 µm	15 µm	20 µm
Mesh 1	526,560	530,780	535,000
Mesh 2	547,580	552,100	556,620
Mesh 3	632,400	636,620	640,840

Figure A2 plots the cross-sectional average velocities at *x* = 0 in the microchannel of the three different meshes from *t* = 0 s to *t* = 1 s, which is calculated by $U_{ave}=\int_{-H_{c}/2}^{H_{c}/2}U(0,y)dy/H_{c}$. Within 1 s, the cross-sectional average velocities show no significant difference for the three different meshes considering the large fluctuations in the viscoelastic EOF. The time-averaged cross-sectional average velocities are plotted in Figure A2d. For the three meshes of the microchannels with different lengths, the time-averaged cross-sectional average velocities are very close. Relative errors of mesh 2 and mesh 3 are calculated compared with mesh 1, as shown in Table A2. The relative errors are all smaller than 5%.

The three different meshes show remarkable consistency in predicting the velocity field in the microchannel. In order to save computational cost, mesh 1 is used to perform other simulations.

**Table A2:** Relative errors of the cross-sectional average velocity of mesh 2 and mesh 3.

Relative Error	10 µm	15 µm	20 µm
Mesh 2	2.93%	0.63%	3.47%
Mesh 3	1.47%	0.83%	0.49%

## Appendix B. Code Validation

A microchannel between two parallel plates was studied to validate the accuracy of the solver in solving the pulsating electric field. As shown in Figure A3, the height of the microchannel is 40 μm, which is the same as the contraction microchannel in this study. A structural mesh with 169,080 cells is created to perform the validation, as shown in Figure A4.

Newtonian fluid is used for the validation, and the parameters are set as follows: *c*_0_ = 0.0001 mM and η_0_ = 0.0121 kg/(m∙s). A pulsating electric potential bias, U = 40 + 16sin(2*f*_E_π*t*) V, is applied along the *x*-axis between edge AC (inlet) and edge BD (outlet), as shown in Figure A3. The intrinsic potential *ξ*_0_ = −110 mV is used for the solid walls (edge AB and edge CD, as shown in Figure A3) of the microchannel. The Helmholtz–Smoluchowski velocity formula [76] is used to validate the code in solving the pulsating electric fields, where the EOF velocity is approximated as *u* = *εξ*_0_*E_x_*/*η*_0_. In this validation, *E_x_* = *U*/*L*, where *L* = 400 μm.

Figure A5 shows the *x*-component velocity (*u_x_*) at the center of the microchannel (0,0) under the DC electric field and AC electric fields with different frequencies. Under the AC electric field, the velocity–time profile shows as a sine wave, which is the same as the electric potential defined. Figure A6 presents the velocity profile in the upper-half cross-section of the microchannel at different times when *f*_E_ = 4 Hz. Figure A7 shows the velocity field at different times when *f*_E_ = 4 Hz. Figure A8 compares the result of the Helmholtz–Smoluchowski velocity formula and the numerical result. The highest relevant difference is less than 0.18%, which validates the accuracy of the solver in solving the pulsating electric field.
